# Effect of Oral Allylnitrile Administration on Cochlear Functioning in Mice Following Comparison of Different Anesthetics for Hearing Assessment

**DOI:** 10.3389/ftox.2021.641569

**Published:** 2021-02-25

**Authors:** Dorien Verdoodt, Sander Eens, Debby Van Dam, Peter Paul De Deyn, Olivier M. Vanderveken, Krystyna Szewczyk, Vera Saldien, Peter Ponsaerts, Vincent Van Rompaey

**Affiliations:** ^1^Department of Translational Neurosciences, Faculty of Medicine and Health Sciences, University of Antwerp, Antwerp, Belgium; ^2^Laboratory of Experimental Hematology, Faculty of Medicine and Health Sciences, Vaccine and Infectious Disease Institute (Vaxinfectio), University of Antwerp, Antwerp, Belgium; ^3^Laboratory of Neurochemistry and Behaviour, Faculty of Pharmaceutical, Biomedical and Veterinary Sciences, University of Antwerp, Antwerp, Belgium; ^4^Department of Neurology and Alzheimer Research Center, University of Groningen and University Medical Center Groningen, Groningen, Netherlands; ^5^Department of Neurology, Memory Clinic of Hospital Network Antwerp (ZNA) Middelheim and Hoge Beuken, Antwerp, Belgium; ^6^Department of Otorhinolaryngology and Head and Neck Surgery, Antwerp University Hospital, Edegem, Belgium; ^7^Department of Anaesthesiology, Antwerp University Hospital, Edegem, Belgium

**Keywords:** anesthesia, auditory brainstem response, DPOAE, allylnitrile, vestibular function

## Abstract

**Background:** Allylnitrile is a compound found in cruciferous vegetables and has the same lethality and toxic effects as the other nitriles. In 2013, a viable allylnitrile ototoxicity mouse model was established. The toxicity of allylnitrile was limited through inhibition of CYP2E1 with trans-1,2-dichloroethylene (TDCE). The allylnitrile intoxication model has been extensively tested in the 129S1 mouse strain for vestibular function, which showed significant HC loss in the vestibular organ accompanied by severe behavioral abnormalities. However, the effect of allylnitrile on auditory function remains to be evaluated. Commonly used anesthetics to conduct hearing measurements are isoflurane and ketamine/xylazine anesthesia but the effect of these anesthetics on hearing assessment is still unknown. In this study we will evaluate the otovestibular effects of oral allylnitrile administration in mice. In addition, we will compare the influence of isoflurane and ketamine/xylazine anesthesia on hearing thresholds.

**Methods and Materials:** Fourteen Coch+/– CBACa mice were randomly allocated into an allylnitrile (*n* = 8) and a control group (*n* = 6). Baseline measurements were done with isoflurane and 1 week later under ketamine/xylazine anesthesia. After baseline audiovestibular measurements, mice were co-administered with a single dose of allylnitrile and, to reduce systemic toxicity, three intraperitoneal injections of TDCE were given. Hearing loss was evaluated by recordings of auditory brainstem responses (ABR) and distortion product otoacoustic emissions (DPOAE). Specific behavioral test batteries for vestibular function were used to assess alterations in vestibular function.

**Results:** Hearing thresholds were significantly elevated when using isoflurane anesthesia compared to ketamine/xylazine anesthesia for all frequencies of the ABR and the mid-to-high frequencies in DPOAE. Allylnitrile-treated mice lacked detectable ABR thresholds at each frequency tested, while DPOAE thresholds were significantly elevated in the low-frequency region of the cochlea and completely lacking in the mid-to high frequency region. Vestibular function was not affected by allylnitrile administration.

**Conclusion:** Isoflurane anesthesia has a negative confounding effect on the measurement of hearing thresholds in mice. A single oral dose of allylnitrile induced hearing loss but did not significantly alter vestibular function in mice. This is the first study to show that administration of allylnitrile can cause a complete loss of hearing function in mice.

## Introduction

Hearing loss and vestibular impairment are among the most common sensory impairments in the human population. Globally, around 466 million people suffer from disabling hearing loss, of which 34 million are children (WHO, 2018). Hearing loss presents a great risk in everyday life due to problems with speech recognition, interpersonal communication, and language acquisition (Ohlenforst et al., 2017) and is listed by the World Health Organization (WHO) as a priority disease for research into therapeutic interventions to address public health needs (WHO, 2013). Recent epidemiological studies have demonstrated that vestibular disorders affect more than 35% of adults aged 40 or older, increasing to almost 50% between the ages of 60 and 69 (Agrawal et al., 2009). Impaired vestibular function causes imbalance, vertigo, and lack of gaze fixation during head movement, often accompanied by nausea and dizziness (Sedo-Cabezon et al., 2014, 2016).

Ototoxic compounds can cause hearing and vestibular loss. Ototoxicity refers to the toxic damage generated by chemical substances in the inner ear, often resulting in cellular degeneration and temporary or permanent functional impairment (Bisht and Bist, 2011). Ototoxic injury is a major cause of the loss of auditory and vestibular function in both humans and animals (Murillo-Cuesta et al., 2010; Oishi et al., 2012). Several 100 chemical agents are thought to be potentially ototoxic and many of them are either pharmaceuticals, such as aminoglycoside antibiotics and loop diuretics, or industrial chemicals, such as nitriles and various solvents (Bisht and Bist, 2011; Sedo-Cabezon et al., 2014). Drug-induced ototoxic injury has been studied in a large variety of species. Human and rodent data indicate the inner ear neurosensory epithelia as the primary targets of ototoxicity, often causing permanent disability due to ablation of the inner ear hair cells (IHC) (Sedo-Cabezon et al., 2014, 2016). Ototoxic damage is typically bilateral and symmetric (Rybak et al., 2009; Schacht et al., 2012; Llorens et al., 2018). Some drug-induced ototoxic insults primarily target the cochleae (cochleotoxic), whereas others are more selective for the vestibular organs (vestibulotoxic) or injure the neurosensory epithelia of both inner ear end organs equally (Schacht et al., 2012). The permanence and severity of toxicity depend not only on the ototoxic drug, but also on the specific dose, route of administration, duration of use, and individual differences in susceptibility to the ototoxicant (Guthrie, 2008; Murillo-Cuesta et al., 2010).

Nitriles are cyano-substituted organic compounds (R-CN) that occur naturally in many cruciferous vegetables and are also frequently used in the chemical and pharmaceutical industries (Boadas-Vaello et al., 2005; Saldana-Ruiz et al., 2012). The ototoxic properties of nitriles have many features in common with those of the aminoglycoside antibiotics and cisplatin, damaging both the auditory and the vestibular system with selectivity for IHC as their primary target (Crofton et al., 1994; Llorens and Dememes, 1994; Saldana-Ruiz et al., 2013; Sedo-Cabezon et al., 2016). The major toxic effects among the nitriles are acute lethality, osteolathyrism, and neurotoxicity (Soler-Martin et al., 2007). The acute lethality that characterizes many nitriles, including allylnitrile, is mainly due to the release of cyanide in the body through metabolism by the alcohol/acetone-inducible isoform of the P450 cytochrome, CYP2E1, as was demonstrated by studies in CYP2E1-null mice (Lewis et al., 1994; Boadas-Vaello et al., 2009; Saldana-Ruiz et al., 2012; Tanii, 2017). CYP2E1 can be pharmacological inhibited by trans-1,2-dichloroethylene (TDCE) limiting the acute toxicity of allylnitrile but leaving the vestibular and cochlear toxicity unchanged. The effect of oral administration of allylnitrile was already studied in 129S1 mice and resulted in a complete loss of vestibular function accompanied by severe behavioral abnormalities due to significant loss of vestibular HC (Boadas-Vaello et al., 2009). However, the effect of allylnitrile on cochlear function remains to be evaluated.

Hearing function is commonly assessed by the Auditory Brainstem Response (ABR) and Distortion Product Otoacoustic Emissions (DPOAE). DPOAE can be recorded non-invasively by inserting a microphone into the ear canal to capture the ear-sound pressure in response to two stimulating tones. These emissions reflect outer hair cell (OHC) integrity and cochlear function (Jimenez et al., 1999; Guimaraes et al., 2004). ABR reflects the electrical responses of both the cochlear ganglion neurons and the nuclei of the central auditory pathway. The ABR consists of five identifiable waves (waves I-V). Wave I represents the response from the spiral ganglion and auditory nerve, while waves II-V originate from the cochlear nucleus, the superior olivary complex and the lateral lemniscus (Akil et al., 2016). As these techniques require the application of subcutaneous needle electrodes and acoustic probes to measure evoked responses or deliver acoustic stimuli, temporary immobilization of the animals is needed. To accomplish this, isoflurane or a mixture of ketamine/xylazine are the most commonly used general anesthetics during auditory testing in small animals. They both act as depressors of the central nervous system and each has specific benefits and costs (Cederholm et al., 2012; Ruebhausen et al., 2012; Sheppard et al., 2018).

Isoflurane is a volatile halogenated inhalation anesthetic that non-competitively blocks glutamatergic N-methyl-D-aspartate (NMDA) receptors. One of the advantages of isoflurane is that it can be regulated precisely and adapted swiftly due to its co-administration with a carrier gas. Furthermore, it is fast-acting and allows for rapid recovery with few residual effects. Despite all these advantages, isoflurane equipment is relatively expensive and all the concomitant waste gas must be vented (Stronks et al., 2010; Ruebhausen et al., 2012; Sheppard et al., 2018). Ketamine also acts as a non-competitive antagonist of NMDA receptors, but in contrast to isoflurane, this is a dissociative anesthetic. It is often co-administered with xylazine, an alpha-2 adrenoreceptor agonist with sedative and antinociceptive effects. The ketamine-xylazine mixture is administered via either intramuscular, intraperitoneal or intravenous injection. However, there are major disadvantages to the use of this anesthetic including increased risk of death and short-lived anesthetic action. Animals typically require several hours for complete recovery (Lima et al., 2012; Ruebhausen et al., 2012; Huang et al., 2017). Contradictory findings have been reported regarding the effects of both general anesthetics on hearing function. Some studies suggest a negative effect of isoflurane on hearing function relative to ketamine/xylazine (Drexl et al., 2004; Kim et al., 2005), while another study found that isoflurane may improve auditory function by augmenting OHC amplification or by protecting against noise-induced hearing loss (NIHL) (Chung et al., 2007).

The aim of this study is to evaluate the effect of oral allylnitrile administration on cochlear function in mice, in addition we will also compare the influence of isoflurane and ketamine/xylazine anesthesia on ABR and DPOAE measurements.

## Methods

### Animals

Male Coch+/– CBACa.129S1(Cg)-Cochtm1.1Stw/Mmjax mice (*n* = 14) were used for this study. Original breeding pairs of the mice were obtained from The Jackson laboratory (034310-JAX) and further bred at the University of Antwerp. The animals were 17–21 weeks old and had an average body weight of 28.9 ± 1.8 g (range 26.8–32.2 g). These mice were used because they show excellent cochlear sensitivity and limited age-related elevation in ABR thresholds. All animal experimental procedures were approved by the Ethics Committee for Animal Experiments of the University of Antwerp (approval No 2017-51) and were in compliance with the European Community Council Directive (2010/63/EU). Mice were housed four-five per cage in standard type III plastic cages with wood shavings as bedding and given water and standard pelleted rodent chow *ad libitum*. Cages were stored in sound-proof rooms at constant room temperature (20–24°Celsius) and humidity (45%). They were maintained on a 12 h/12 h light–dark cycle. Tests were carried out during the light phase of the light-dark cycle.

### Study Design

**Figure 1:**
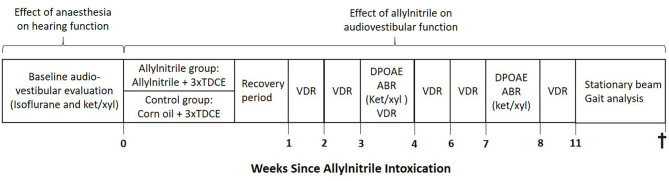
Schematic overview of the study design.

At the start of the experiment, animals were randomly allocated into two groups: an allylnitrile (*n* = 8) and control group (*n* = 6). Baseline audiovestibular evaluation was performed in both groups of mice to examine the pre-intoxication hearing status and vestibular function. To enable direct comparison of anesthesia effects on hearing sensitivity and cochlear OHC function, baseline ABR and DPOAE thresholds were assessed in all mice (*n* = 14) under both injection (ketamine/xylazine) and inhalation (isoflurane) anesthetics. After baseline testing, experimental induction was completed via systemic exposure of mice to either allylnitrile and 3x TDCE (allylnitrile group), or vehicle (corn oil) only (control group), as previously performed (Saldana-Ruiz et al., 2013). One animal died after allylnitrile administration. After a 1 week recovery period following allylnitrile- or sham exposure, all animals remaining were repeatedly tested for hearing and vestibular function at various predetermined points in time. ABR and DPOAE thresholds were evaluated 3 and 7 weeks after ototoxic injury, using ketamine/xylazine anesthetics. Vestibular function was indirectly assessed 1, 2, 3, 4, 6, and 8 weeks after ototoxic insult through using the vestibular dysfunction index (VDI), and 11 weeks post-dosing through gait analysis and stationary beam testing. At the end of functional testing, all mice were sacrificed by an overdose of pentobarbital (120 mg/kg body weight). An overview of the study design is given in Figure 1.

### Anesthetics

Mice were tested with isoflurane anesthesia first, and after 1 week of recovery ABR and DPOAE measurements were done under ketamine/xylazine anesthesia. DPOAE measurements were done before ABR assessment to avoid reduced DPOAE responses, measurements were conducted consecutively without awakening of the animal. Mice were anesthetized with either isoflurane (4% for induction and 1.5–2% for maintenance) or an intraperitoneal (i.p.) injection with a ketamine (100 mg/kg body weight) and xylazine (20 mg/kg body weight) mixture. Hearing assessment was started 15 min after ketamine/xylazine injection. After anesthesia induction, mice were individually placed in a sound-attenuating chamber (Industrial Acoustic Company, North Aurora, IL, USA) on a homeothermic heating pad system (Harvard Apparatus, Holliston, MA, USA) to maintain constant body temperature (37 ± 0.5°C). Prior to each recording session, ophthalmic ointment (Duratears, Alcon) was applied to the eyes to prevent corneal drying.

### Allylnitrile Administration

Mice received a single-dose of allylnitrile peroral (p.o.) in 6 ml/kg of corn oil at 0 or 1.0 mmol/kg and three injections of TDCE i.p. in 6 ml/kg of corn oil at 0 or 100 mg/kg, administered 30 min before and 6 and 24 h after administration of allylnitrile. In the days following allylnitrile intoxication, all animals were daily weighed and visually inspected for 4 weeks and at least twice a week afterwards. Nutritional support and supplemental hydration were provided via mush and subcutaneous injections of 0.9% NaCl, respectively. Score sheets for welfare assessment and evaluation of weight loss were used, and mice were euthanized if they met the criteria of the ethical limits of suffering but in our study none of the mice reached humane endpoint. Allylnitrile, TDCE and corn oil were purchased from Merck Sigma-Aldrich (Overijse, Belgium).

### Hearing Evaluation

#### Auditory Brainstem Response

Evoked responses were recorded using disposable subcutaneous needle electrodes (28G) positioned over the vertex of the skull (active electrode), the left mastoid (reference electrode), and the right hindlimb (ground electrode). Electrode placement was manipulated until an impedance of no higher than 2 kOhm was observed. Evoked potentials were measured after administration of frequency-specific sound stimuli through a free-field electrostatic speaker placed 10 cm in front of the animal's head. BioSig32 software (Tucker-Davis Technologies, Alachua, FL, USA) was used to generate tone burst stimuli of 2 ms in length with a gate of 1 ms at frequencies 2, 4, 8, 16, and 32 kHz in 5 dB steps starting at 80 dB SPL down to a minimum SPL of 10 dB. A stimulus repetition rate of 32 per second was used and 800 trials were recorded for each frequency to obtain a good averaged response. Since potentials have very low amplitude, averaging of 800 stimulations was essential to differentiate the typical waveform of the evoked potential from background noise. Auditory Brainstem Response (ABR) thresholds were defined as the lowest stimulus level at which any reproducible ABR waveform could be reliably observed in the evoked response at appropriate latencies upon visual inspection and was determined by comparing the ABR waveforms with several suprathreshold ABRs. Threshold analyses were performed via offline analysis of stored waveforms, and the thresholds obtained for each frequency were verified at least twice by the same experimenter. In addition, amplitudes of wave I were assessed at 8 and 16 kHz. At the other frequencies, this parameter was not analyzed because under isoflurane anesthesia it was difficult to identify wave I at 2, 4, and 32 kHz. Upon completion of testing, needle electrodes were removed, lidocaine (Xylocaine, 2%, Pfizer, Puurs, Belgium) was applied topically, and animals were moved individually to heated cages and monitored until complete recovery. Duration of testing was approximately 30 min per animal. To enable statistical analysis and calculations of the mean, unobtainable ABR thresholds at our equipment's limits of 80 dB SPL were defined as 85 dB SPL.

#### Distortion Product Otoacoustic Emissions

For Distortion Product Otoacoustic Emissions (DPOAE) measurements, all anesthetized mice were placed on their left flank under a device securely holding an acoustic probe tightly fitted into the right external auditory canal. Two tones (f1 and f2) were administered simultaneously in the right ear only via a close-field method using BioSig32 software (Tucker-Davis Technologies, Alachua, FL, USA). DPOAE responses (2f1–f2) were measured by a microphone (Nexus Microphone Conditioner, Bruel and Kjaer, Naerum, Denmark) that was calibrated before testing over a frequency range from 5 to 32 kHz, more specifically at 5.3, 6.1, 7.0, 8.0, 9.2, 10.6, 12.1, 13.9, 16.0, 18.4, 21.1, 24.3, 27.9, and 32.0 kHz. The primary tone ratio f2/f1 was set to 1.22. DPOAE responses were evoked by a non-symmetric DPOAE protocol, using unequal primary tone stimulus intensities (i.e., L1 > L2). Five intensity levels were presented with L1 going from 70 to 30 dB SPL and L2 = L1 – 10 dB SPL. Duration of testing was approximately 20 min per animal. To enable statistical analysis and calculations of the mean, unobtainable DPOAE thresholds at our equipment's limits of 70 dB SPL were defined as 80 dB SPL.

### Vestibular Evaluation

#### Vestibular Dysfunction Index

The VDI is a behavioral test battery that has been successfully used to assess the loss of vestibular function in rats and mice following systemic exposure to ototoxic nitriles, including allylnitrile (Boadas-Vaello et al., 2017). The battery includes three measures of spontaneous motor behavior and three vestibular reflexes that are rated 0 (normal behavior) to 4 (maximal deficit in behavior). By adding up the rating scores for all six behavioral tests, a summary score (VDI, 0–24) is obtained indicative of vestibular dysfunction. For the observation of spontaneous motor behavior, mice were placed for 1 min in an empty rat cage and were rated from 0 to 4 for circling, retropulsion, and abnormal head movements. Circling is defined as spontaneous stereotyped circling or tail-chasing locomotion. Retropulsion consists of backwards displacement, and abnormal head movements are defined as exaggerated up and down movements of the head, often referred to as head bobbing. Next, for the evaluation of vestibular reflexes, the mice were removed from the rat cage and were rated from 0 to 4 for the tail-lift reflex, contact inhibition of the righting reflex, and the air-righting reflex. For the tail-lift reflex, mice were lifted by the base of their tail. As a result, normal mice extend their body and fore paws as a “landing” response. Mice with impaired vestibular function, however, curl their bodies ventrally, crawling up toward their tails. For contact inhibition of the righting reflex, light pressure was applied on a transparent plastic board in contact with mice that are flipped supine on a tabletop. Healthy mice quickly right themselves, whereas mice with vestibular dysfunction remain on their back with their feet up and walk with respect to the ventrally positioned plastic board. For the air righting reflex, mice were dropped supine from a height of 30 cm onto a foam cushion. Normal mice right themselves in the air during the fall, whereas vestibular-deficient mice do not.

#### Gait Topography Analysis and Stationary Beam Test

To evaluate the coordination of motor function, indicative for vestibular function, mice were subjected to gait analysis and a stationary beam test.

The stationary beam test setting used to evaluate balance and motor functions consisted of a wooden beam (diameter: 25 mm, length: 110 cm) covered with a layer of masking tape to provide a firmer grip. The beam was divided into 11 segments and placed at a height of 38 cm above a cushioned floor. Beam ends were shielded with pieces of cardboard to prevent mice from escaping. Testing started by placing the mice in the middle of the beam. The number of segments crossed (four-paw criteria), the latencies before falling, and the number of falls were measured for four trials with a cut-off period of 1 min per trial and an intertrial interval of 10 min. Mice were not trained for this behavioral test.

Gait characteristics (stride length and track width) were assessed by applying ink to the animals' hind paws and letting them walk on a strip of paper down a brightly lit alley (4.5 cm wide, 40 cm long) toward a dark goal box, leaving a permanent record of its footprints.

### Euthanasia

At the end of the experiment mice were euthanized using an overdose of pentobarbital (120 mg/ml).

### Statistical Analysis

To analyse the effects of allylnitrile, for each test parameter, a separate Linear Mixed Model (LMM) was constructed. The ABR threshold, DPOAE threshold, and VDR score, were used as dependent variables, whereas time and treatment were listed as categorical fixed effects. In addition, the interaction term time^*^treatment was added. This interaction term tests the null hypothesis that the effects of time and treatment are independent. If the interaction term is significant, this indicates that the change over time differed between the control and allylnitrile group. Furthermore, LMMs were fitted for evaluation of significant time effects within each treatment group. For ABR and DPOAE data, LMMs were always fitted for each frequency separately. In case of significant time effects in the allylnitrile group, *post-hoc* pairwise comparisons were carried out with Tukey's correction for multiple testing. Statistically significant group differences for all tests were evaluated via separate Mann-Whitney U tests. Non-parametric Wilcoxon signed-rank testing was performed for direct comparison of anesthesia effects on hearing sensitivity and cochlear OHC function in the same mice. All data are represented as mean ± standard deviation (SD). Data were considered statistically significant as ^*^p ≤ 0.05, ^**^p ≤ 0.01, and ^***^p ≤ 0.001. Statistical analyses were computed using IBM SPSS Statistics 25.0 software and R software. GraphPad Prism 8.0.2 was used for the generation of graphs.

## Results

### Effect of General Anesthetics on Hearing Function

#### Distortion Product Otoacoustic Emissions

**Figure 2:**
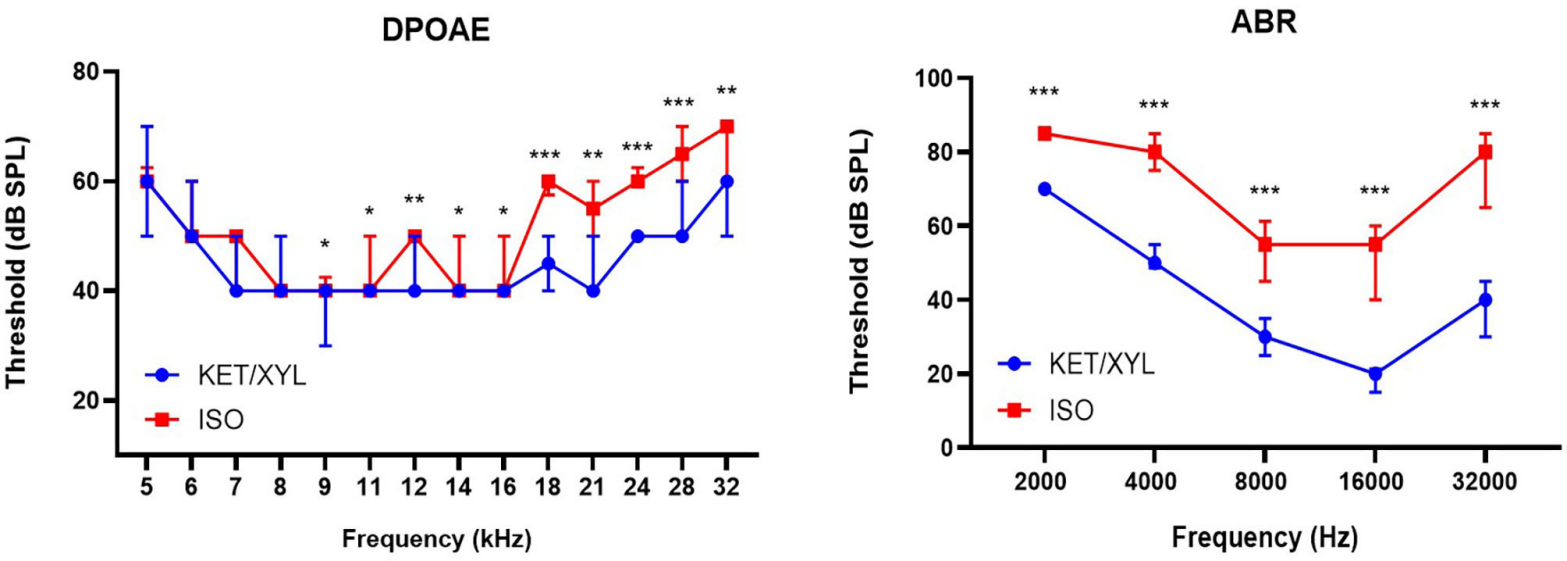
Effect of general anesthetics on hearing sensitivity and cochlear OHC function in mice (*n* = 14). DPOAE thresholds (Distortion Product Otoacoustic Emissions) measured under isoflurane (ISO) and ketamine/xylazine (KET/XYL) at frequency range 5–32 kHz (left). Thresholds of ABR (Auditory Brainstem Responses) were measured at five frequency levels: 2, 4, 8, 16, and 32 kHz (right). Data are presented as median with interquartile range. *indicates *p* ≤ 0.05; **indicates *p* ≤ 0.01; ***indicates *p* ≤ 0.001.

Close-field DPOAE responses were recorded over a range of 14 frequencies from 5.3 to 32.0 kHz. Mice under isoflurane anesthesia showed significantly elevated DPOAE thresholds in the mid-to-high frequency region of the cochlea (≥9.2 kHz) relative to mice anesthetized with ketamine/xylazine (Wilcoxon signed-rank test, *p* ≤ 0.05 for all frequencies). In the low-frequency region (≤ 8.0 kHz), no significant changes in DPOAE thresholds were observed between both anesthetics (Figure 2).

#### Auditory Brainstem Response

Free-field ABR recordings were recorded over a range of five frequencies from 2 to 32 kHz (*n* = 14). Across the entire frequency range tested, ABR thresholds obtained under isoflurane were significantly elevated relative to those obtained under ketamine/xylazine anesthesia (Wilcoxon signed-rank test, *p* ≤ 0.001 for all frequencies). On average, thresholds were elevated by more than 13 dB at 2 kHz, and by more than 25 dB at 4, 8, 16, and 32 kHz, with a maximal average threshold shift of 35.7 dB at 32 kHz (Figure 2).

**Figure 3:**
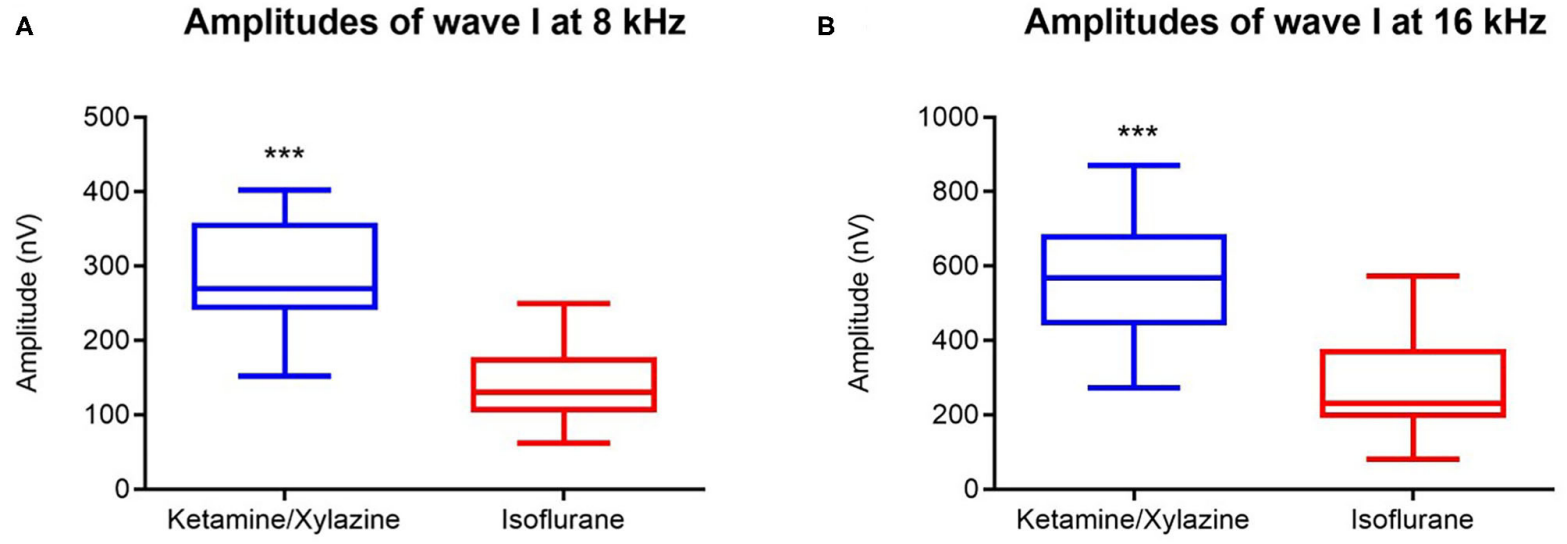
Effect of general anesthesia on wave I amplitude in mice (*n* = 14). Wave I amplitudes measured under isoflurane (red boxplot) and ketamine/xylazine anesthesia (blue box plot) at 8000 Hz **(A)** and at 16000 Hz **(B)** (for both *n* = 14). Data are represented by box plots. ***indicates *p* ≤ 0.001.

Amplitudes of wave I, representing the response of the spiral ganglion neurons and the auditory nerve, were significantly reduced under isoflurane anesthesia compared to ketamine/xylazine anesthesia (Wilcoxon signed-rank test *p* ≤ 0.001, 8kHz and 16 kHz). Average reduction in wave I amplitude was 143.9 nV at 8 kHz and 227.4 nV at 16 kHz (Figures 3A,B).

### Effect of Allylnitrile on Vestibular Function

The results of the allylnitrile administration experiment were established with data obtained on six out of eight mice treated with 1.0 mmol/kg allylnitrile, and five out of six control mice. One animal of the allylnitrile group died hours after the final (third) injection of TDCE, whereas the other was left out since allylnitrile was maladministered. At baseline hearing testing, prior to allylnitrile intoxication, a control animal died only minutes after completion of the ABR recording, most likely as a consequence of anesthetic induction with ketamine/xylazine anesthesia.

#### Vestibular Dysfunction Rating

**Figure 4:**
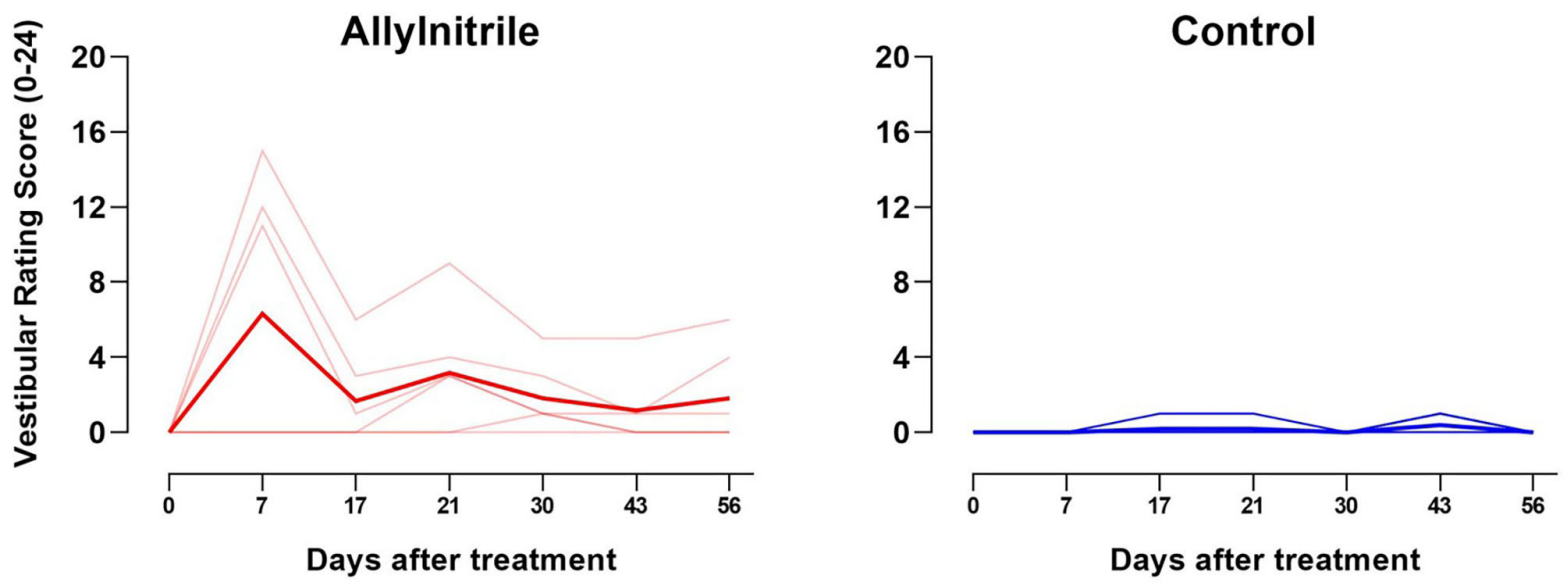
Effect of allylnitrile on rating scores for vestibular function. Vestibular dysfunction rating (VDR) scores for allylnitrile (red, *n* = 6) and control (blue, *n* = 5) mice are shown individually (transparent lines), as well as the group means (solid lines). The graph shows the sum of all six rating scores from 0 (minimal deficit in behavior) to 24 (maximal deficit in behavior) for allylnitrile-treated (left) and control (right) mice. One week after allylnitrile, allylnitrile-treated mice revealed increased vestibular rating scores to intermediate values (i.e., 11–15), where after rating scores progressively declined to values just above or in the control range.

A LMM was fitted to test whether the change in vestibular rating scores over time differed between the control (*n* = 5) and allylnitrile (*n* = 6) group, revealing a significant time x treatment interaction (F6,54 = 3.59, *p* = 0.005). Baseline examination of vestibular function revealed no behavioral abnormalities in both groups of mice (all baseline scores = 0). One week after allylnitrile administration, moderate behavioral abnormalities were observed in three out of six allylnitrile-treated mice with vestibular rating scores increasing to intermediate values (i.e., 11–15). Over the next weeks, however, rating scores of the affected subjects progressively declined to values just above or within the control range (i.e., 1–6). Behavioral scorings of control mice remained within the normal control range (0–2) across all points in time, as well as the vestibular rating scores of the other three non-affected allylnitrile-treated mice. At day 30 only, Mann-Whitney U testing revealed significantly elevated vestibular rating scores. This data is represented in Figure 4.

Separately fitted LMMs for each treatment group revealed a significant effect of time for the allylnitrile group (*p* = 0.003), but not for the control group (*p* = 0.184). Within the allylnitrile group, pairwise comparisons with baseline were found to be significant for day 7 (*p* < 0.01), but not for all other post-dosing experimental times. Between day 7 and day 17, vestibular rating scores of allylnitrile-treated mice decreased significantly (*p* = 0.013).

#### Gait Topography Analysis and Stationary Beam Test

**Figure 5:**
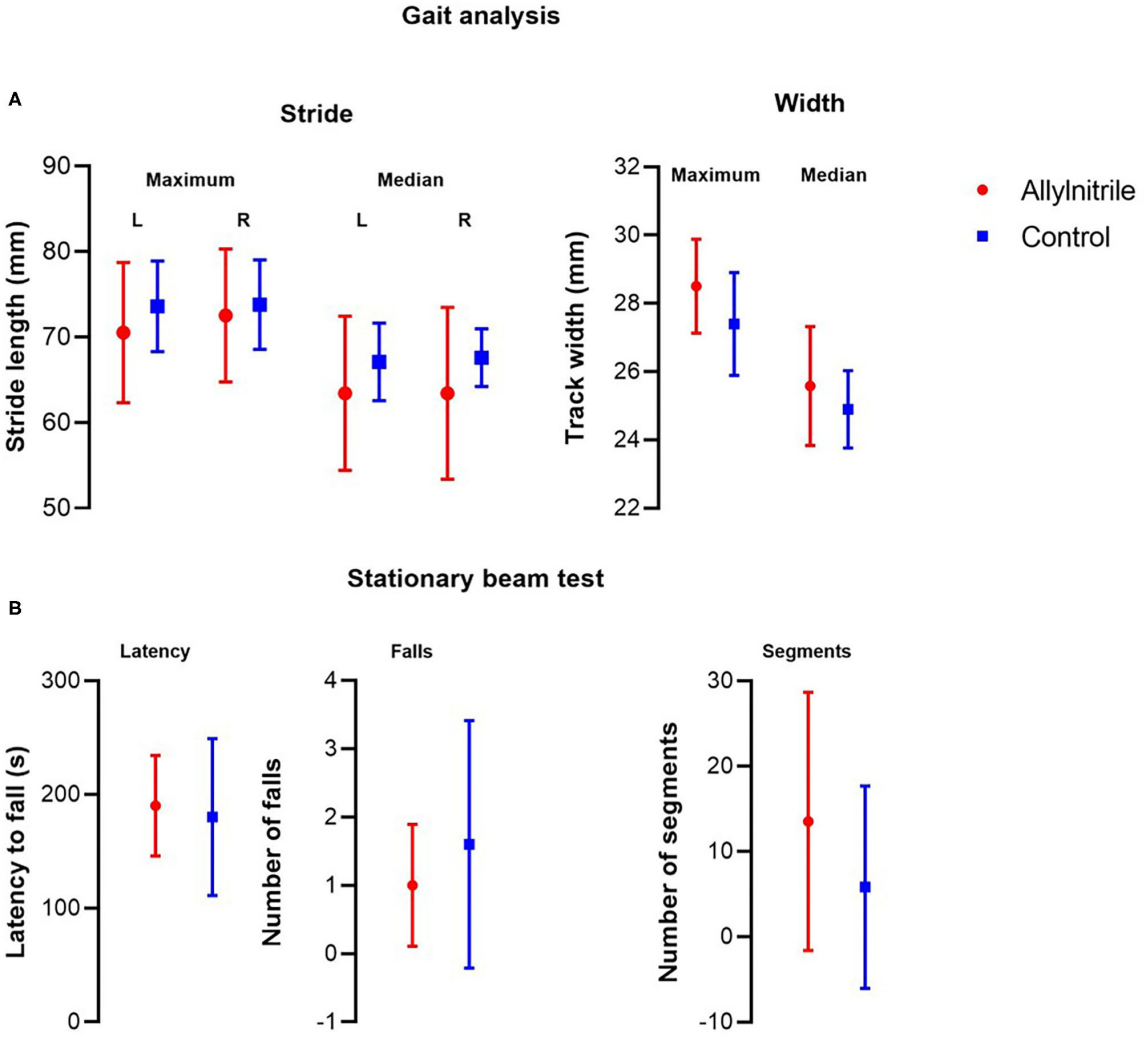
Effect of allylnitrile on gait topography and stationary beam test performance. To evaluate balance and coordination of motor function, allylnitrile (red, *n* = 6) and control (blue, *n* = 5) mice were examined for performance in gait topography analysis and stationary beam test. **(A)** Gait analysis included measurements of stride length (maximum and median, left and right) and track width (maximum and median). **(B)** For stationary beam testing, the latencies before falling, the number of falls, and the segments crossed were measured for four trials. For all tests, no significant group differences in performance were observed. Data are represented as mean ± SD. Statistical analysis consisted of multiple Mann-Whitney U tests. L, left; R, right.

Analysis of gait topography revealed no statistically significant group differences in maximum (left, *p* = 0.410; right, *p* = 0.784) and median (left, *p* = 0.273; right, *p* = 0.361) stride length, as well as in maximum (*p* = 0.304) and median (*p* = 0.518) track width (Figure 5A). Coordination of motor function and balance were tested via the stationary beam test. Stationary beam testing revealed no statistically significant group differences in the number of segments covered (*p* = 0.052), the total number of falls (*p* = 0.705), and the latencies to fall (*p* = 0.852) (Figure 5B).

### Effect of Allylnitrile on Hearing Function

#### Auditory Brainstem Response

To evaluate hearing loss associated with allylnitrile intoxication, free-field ABR thresholds across a broad frequency range were evaluated at three distinct timepoints: once prior to allylnitrile intoxication (baseline) and twice afterwards, at 22- and 50-days post allylnitrile (22/50 DPA). For all these tests ketamine/xylazine anesthesia was used. LMMs were fitted for each frequency separately to test whether the change in ABR threshold over time differed between the two treatment groups, revealing significant interactions between time and treatment at all frequencies (*p* ≤ 0.001 for all frequencies, see Supplementary Table 1A).

**Figure 6:**
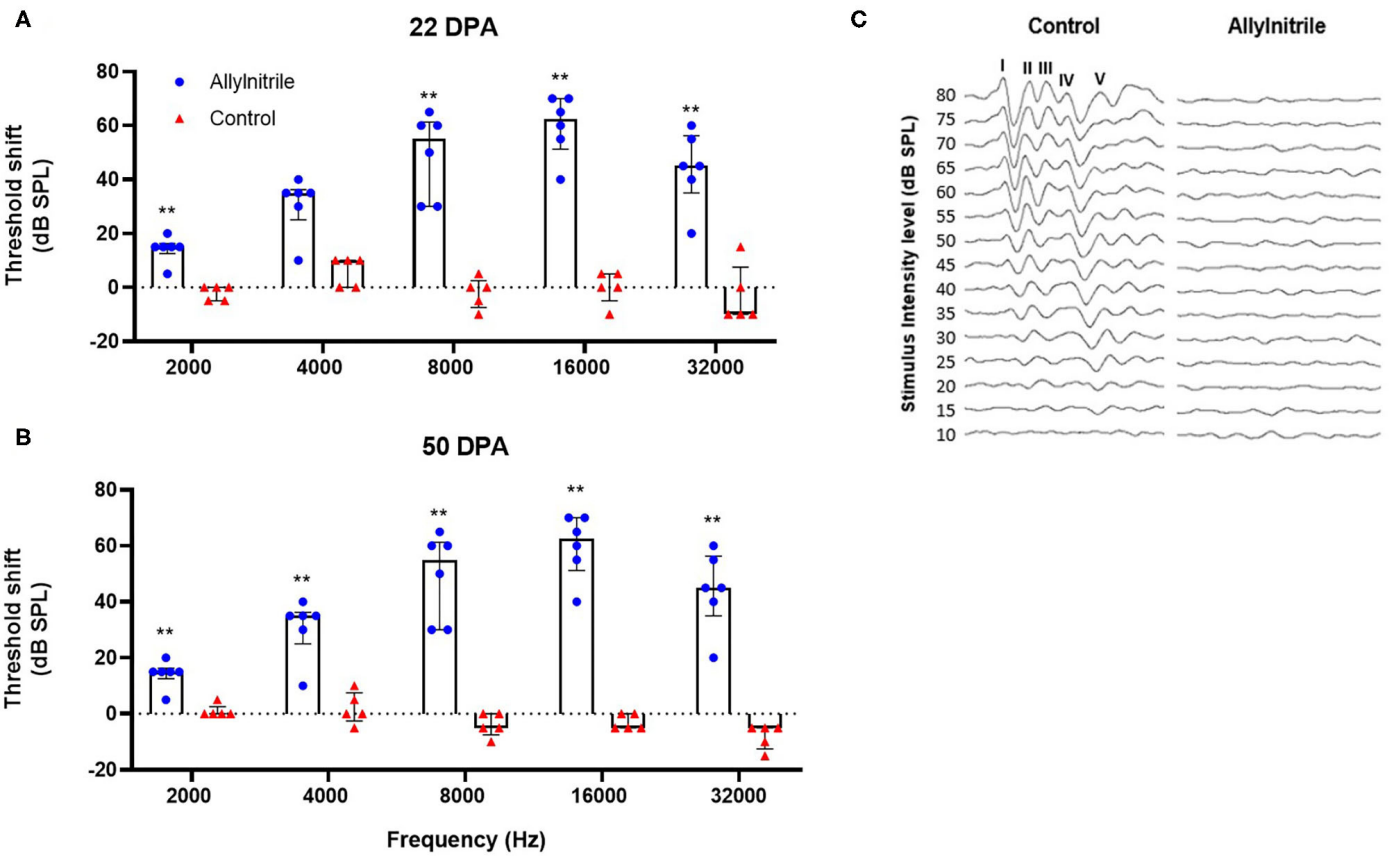
Effect of allylnitrile on hearing sensitivity. **(A)** ABR (Auditory Brainstem Response) threshold shifts of allylnitrile (blue dots, *n* = 6) and control (red dots, *n* = 5) mice at 22 DPA (days post allylnitrile) Thresholds of ABR were measured at five frequency levels: 2, 4, 8, 16, and 32 kHz. **(B)** ABR threshold shifts in the same mice at the same frequencies at 50 DPA. **(C)** Averaged ABR waveform tracings from a control corn oil and allylnitrile-treated mice at 50 DPA. Tracings were measured in response to 16-kHz tone burst stimuli, starting at 80 dB SPL down to a minimum SPL of 10 dB. In the control mouse, tracings consisted of five identifiable waveforms, labeled I-V, whereas all the waves were completely absent in the allylnitrile-treated mouse. The circles and triangles represent the data of the individual mice, the bars represent each group median and interquartile range. Statistical analysis consisted of multiple Mann-Whitney U tests, **indicates *p* ≤ 0.01.

Mann-Whitney U tests were performed for each timepoint and each frequency separately to test for statistically significant group differences in ABR thresholds. At baseline, frequency-threshold curves of allylnitrile-treated (*n* = 6) and control (*n* = 5) mice were nicely overlapping with no significant group differences (*p* > 0.05 for all frequencies, see Supplementary Table 1A). All baseline ABR waves exhibited normal morphology displaying the five standard peaks (I-V). At 22 DPA, allylnitrile-treated mice showed significantly elevated ABR threshold shifts relative to controls at all frequencies tested (*p* ≤ 0.01 for all frequencies, Figure 6A, see Supplementary Table 1A). Similarly, at 50 DPA, significant group differences were observed at all frequencies (*p* ≤ 0.01 for all frequencies, Figure 6B, see Supplementary Table 1A). LMMs were fitted for each frequency separately to test if there were significant differences in ABR threshold within each treatment group across all three points in time. Main effects of time were significant for all frequencies in the allylnitrile group (*p* ≤ 0.001 for all frequencies), but never in the control group (*p* > 0.05 for all frequencies, see Supplementary Table 1B). *Post-hoc* pairwise comparisons of ABR thresholds using Tukey's correction for multiple testing were carried out for all frequencies in the allylnitrile group. At all frequencies, baseline recordings differed significantly from both follow-up measurements (22/50DPA) (*p* ≤ 0.001 for all frequencies). In contrast, thresholds never significantly differed between the two follow-up measurements (*p* > 0.05 for all frequencies, see Supplementary Table 1B). Figure 6C shows averaged ABR waveform tracings of control and allylnitrile-treated mice. In the control tracings, ABR waveforms remained present until 15 dB SPL, whereas in the tracings of allylnitrile-treated mice, all five waveforms, labeled I-V in the figure, were completely absent at each SPL. The ABR threshold was defined as the lowest stimulus level for which any reproducible ABR waveform in the evoked response can be reliably observed.

#### Distortion Product Otoacoustic Emissions

**Figure 7:**
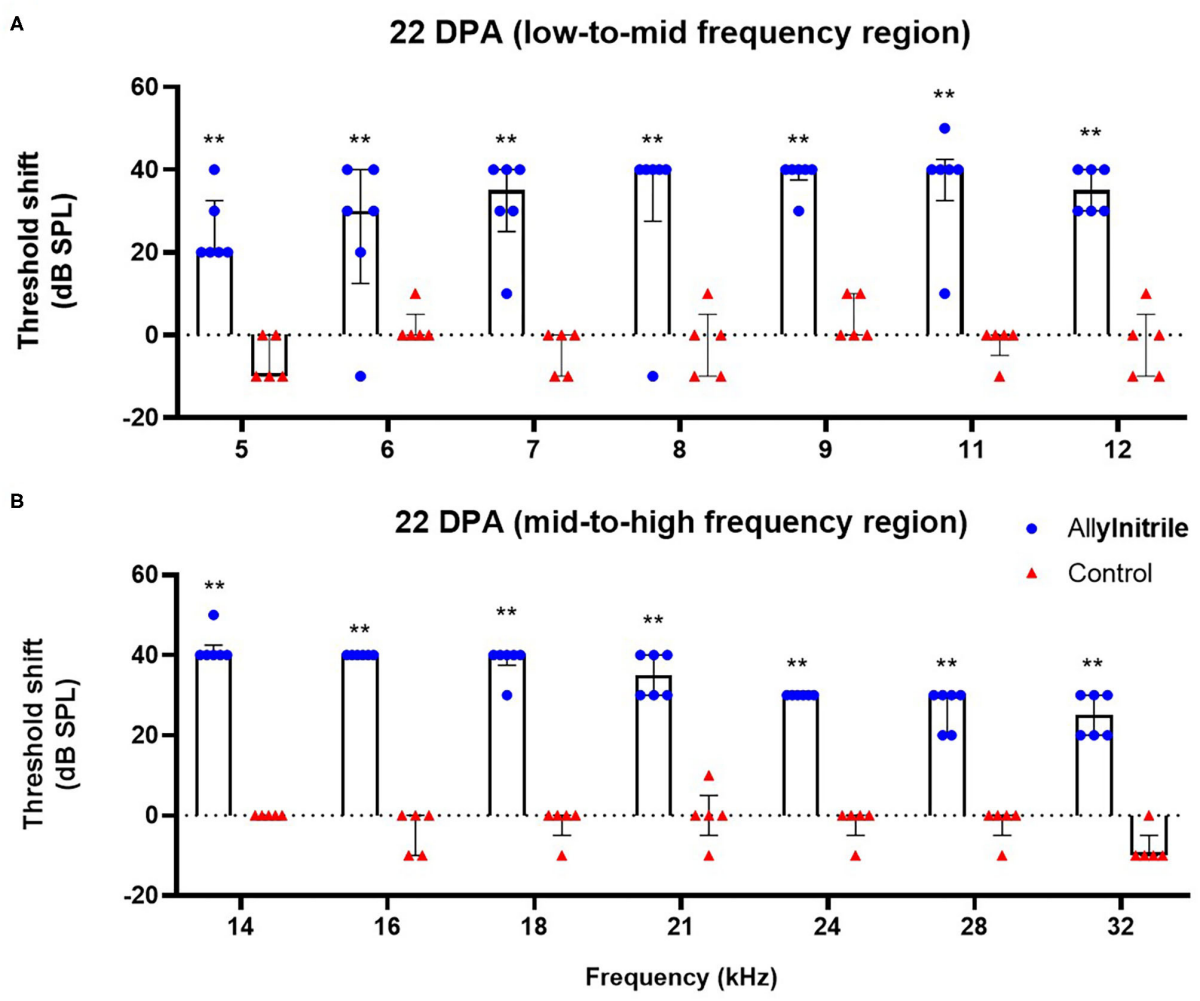
Effect of allylnitrile on cochlear OHC function at 22 DPA. **(A)** DPOAE (Distortion Product Otoacoustic Emissions) threshold shifts of allylnitrile (blue circles, *n* = 6) and control (red triangles, *n* = 5) mice plotted as a function of stimulus f2 in the low-to-mid frequency at 22 DPA (days post allylnitrile). Thresholds were recorded at 14 frequency levels ranging from 5 to 32 kHz. **(B)** DPOAE threshold shifts of allylnitrile (blue circles *n* = 6) and control (red triangles, *n* = 5) mice plotted as a function of stimulus f2 in the mid-to-high frequency at 22 DPA. The circles and triangles represent the data of the individual mice, the bars represent each group median and interquartile range. Statistical analysis consisted of multiple Mann-Whitney U tests, **indicates *p* ≤ 0.01.

**Figure 8:**
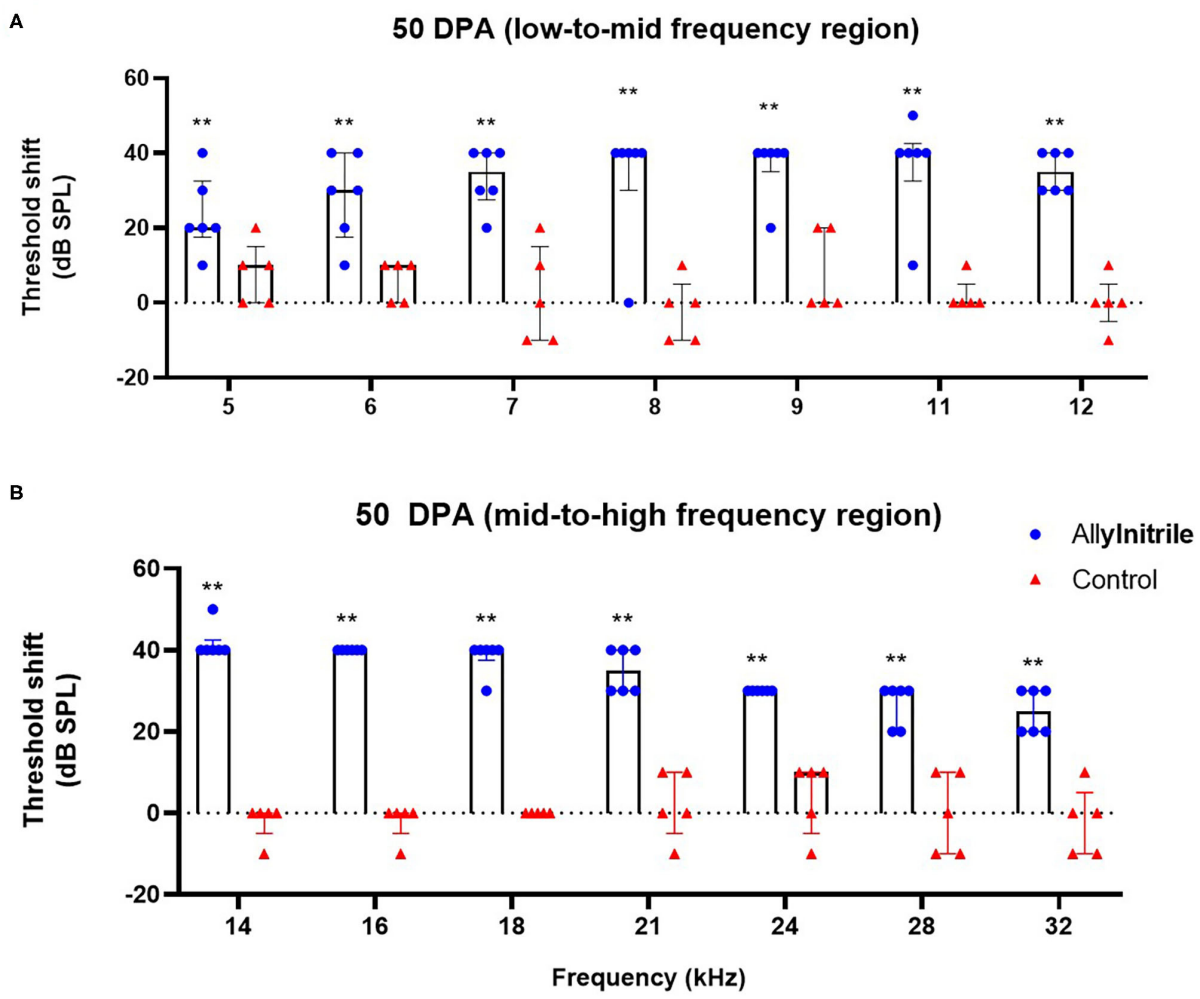
Effect of allylnitrile on cochlear OHC function at 50 DPA. **(A)** DPOAE (Distortion Product Otoacoustic Emissions) threshold shift of allylnitrile (blue circles, *n* = 6) and control (red triangles, *n* = 5) mice plotted as a function of stimulus f2 in the low-to-mid frequency at 50 DPA (days post allylnitrile). Thresholds were recorded at 14 frequency levels ranging from 5 to 32 kHz. **(B)** DPOAE threshold shifts of allylnitrile (blue circles *n* = 6) and control (red triangles, *n* = 5) mice plotted as a function of stimulus f2 in the mid-to-high frequency at 50 DPA. The circles and triangles represent the data of the individual mice, the bars represent each group median and interquartile range. Statistical analysis consisted of multiple Mann-Whitney U tests, **indicates *p* ≤ 0.01.

To investigate the ototoxic effects of acute allylnitrile exposure on cochlear OHC function, close-field DPOAE thresholds of the right ear were examined at 14 frequencies ranging from 5 to 32 kHz. Similar as for ABR, mice were evaluated at baseline and twice afterwards, at 22 and 50 DPA, respectively. LMMs revealed significant interactions between time and treatment at all frequencies (*p* ≤ 0.01 at 6.1 kHz and *p* ≤ 0.001 for all other frequencies, see Supplementary Table 2A). At baseline, DPOAE thresholds did not differ significantly between allylnitrile (*n* = 6) and control mice (*n* = 5) (Mann-Whitney U test, *p* > 0.05 for all frequencies, see Supplementary Table 2A). In contrast, at 22 and 50 DPA, allylnitrile-treated mice displayed significantly elevated DPOAE threshold shifts relative to controls at nearly all frequencies tested (Mann-Whitney U test, *p* ≤ 0.01 for nearly all frequencies, Figures 7A,B, 8A,B, see Supplementary Table 2A). For both points in time, allylnitrile-treated mice lacked detectable DPOAE thresholds in the mid-to high-frequency region of the cochlea (≥12.1 kHz), whereas significantly elevated thresholds were confined to the low-to mid-frequency region (5.3–11.0 kHz). Main effects of time were significant for all frequencies in the allylnitrile group (*p* ≤ 0.001 for all frequencies), and for 5.3 kHz in the control group (*p* ≤ 0.01, see Supplementary Table 2B). Similar as for ABR, *post-hoc* pairwise comparisons of DPOAE thresholds in the allylnitrile group revealed significant differences between baseline and 22/50 DPA (*p* ≤ 0.001 for all frequencies), but not between both follow-up measurements (*p* > 0.05 for all frequencies, see Supplementary Table 2B).

## Discussion

### Effects of Anesthesia on Hearing Measurements

#### Effects of Anesthesia on Hearing Sensitivity

The data obtained in this study demonstrate that hearing thresholds obtained under isoflurane anesthesia were significantly elevated relative to those obtained under ketamine/xylazine anesthesia at all frequencies tested. Our results obtained with Coch+/– CBACa.129S1(Cg)-Cochtm1.1Stw/Mmjax mice are in line with a study performed on C57Bl/6J and C129/SvEv mice where elevated ABR thresholds under isoflurane were significantly worse than under ketamine/xylazine anesthesia at the most sensitive region of hearing (16 kHz) (Cederholm et al., 2012). A study performed in rats also showed significantly elevated ABR threshold among all frequencies tested (ranging from 8 to 32 kHz) under isoflurane anesthesia relative to ketamine/xylazine anesthesia with the greatest threshold shift found in the region between 12 and 24 kHz (Ruebhausen et al., 2012). In contrast, Kim and colleagues found no differences between ABR baseline recordings using BALB/c mice (Kim et al., 2012). Our study revealed a significant reduction in wave I amplitude when isoflurane anesthesia was used. Reduced wave I amplitudes are associated with a reduction in auditory nerve fibers due to aging or noise-exposure, also called synaptopathy (Mehraei et al., 2016). Exposure to moderate noise can cause temporary threshold shifts in mice, while the amplitude of wave I can be permanently reduced. Histological analysis of the cochleae of these mice has demonstrated that hair cells are intact but the number of ribbon synapses and afferent fiber terminals are significantly reduced (Liu et al., 2019). Reduction of wave I under isoflurane anesthesia indicates that isoflurane has an effect on the synapse between the inner ear cells and the afferent fiber terminals of the spiral ganglion neurons.

#### Effects of Anesthesia on Outer Hair Cell Function

Our results demonstrate that, in the mid-to high range tones of the cochlea (i.e., 9.2–32 kHz), DPOAE thresholds obtained under isoflurane anesthesia were significantly elevated compared to those obtained under ketamine/xylazine anesthesia. Similarly, Kim and colleagues observed poorer DPOAE thresholds across all frequencies in mice anesthetized with isoflurane (Kim et al., 2012). Data obtained by Sheppard colleagues in Sprague-Dawley rats also reported that usage of isoflurane anesthesia could significantly reduce DPOAE amplitudes relative to ketamine/xylazine (Sheppard et al., 2018). In contrast, Cederholm and colleagues observed no significant differences in DPOAE thresholds between both anesthetics (Cederholm et al., 2012).

#### Influence of Anesthesia on Cochlear Function

The exact mechanisms underlying the differential actions of isoflurane and ketamine/xylazine on hearing and cochlear OHC function are unclear and remain widely discussed. Previous studies have indicated that isoflurane potentiates glycine receptor currents, the GABA receptor (GABAR) and the kainite selective glutamate receptor *in vitro*, whereas isoflurane inhibits the nicotinic acetylcholine receptor (nAChR) (Cederholm et al., 2012). Moreover, it has been demonstrated that isoflurane has an effect on NMDA receptors. All these receptors are expressed in the mammalian inner ear and have an important role in the neurotransmitter systems regarding hearing function. In contrast, ketamine only has a large effect on the NMDA receptor by acting as a non-competitive antagonist and a small inhibitory effect on the nAChR (Yamakura et al., 1993; Hirota and Lambert, 1996, 2011). Other studies have found evidence that isoflurane interacts with voltage-gated Na+ and Ca2+ channels and can inhibit neurotransmitter release (Herring et al., 2009). Whereas ketamine has been shown to decrease blood flow to the inferior colliculus, isoflurane use may increase blood flow to the brainstem or impair blood flow in the cochlea (Boarini et al., 1984; Sheppard et al., 2018). Experimental differences regarding setup, protocol, dosing, ketamine/xylazine injection location as well as species-, strain- or age-related differences—at least partially—explain the contradictory results available in literature.

### Effect of Allylnitrile on Vestibular Function

#### Vestibular Dysfunction Rating

The test batteries for vestibular function used in our study demonstrate little to no vestibular impairment resulting from allylnitrile-induced ototoxic injury. A similar decrease in VDIs over time, as in the present study, has been observed in rats and mice after IDPN ototoxicity (Llorens et al., 1993; Boadas-Vaello et al., 2017). Very little is known regarding the cellular and molecular mechanisms involved in functional recovery of vestibular function during washout after acute ototoxicity. One study has reported experimental evidence of vestibular HC regeneration after ototoxic injury (Rubel et al., 1995; Sergi et al., 2003) although this is highly unlikely to provide significant functional recovery. Others have suggested that CNS compensation may partly account for behavioral recovery (McCall and Yates, 2011).

#### Gait Topography and Stationary Beam

Additional tests of balance and motor coordination confirmed the preserved vestibular function in allylnitrile-treated mice 11 weeks post-dosing. Increase in the surface of support is a typical compensatory effect of vestibular impairment in quadruped animals (Boadas-Vaello et al., 2005, 2017; Tighilet et al., 2015). However, gait topography analysis revealed no significant group differences in stride length or track width following allylnitrile exposure. In addition, also stationary beam testing demonstrated no significant group differences, although there was an almost/borderline significant (*p* = 0.052) difference in the number of segments covered, with allylnitrile-treated mice crossing a higher number of segments. However, this is opposite to what we would expect.

#### Differences Between Mouse Strains

The preservation of normal vestibular function in our study is discordant with the moderate to severe vestibular dysfunction observed in the initial study on the 129S1 mouse strain (Saldana-Ruiz et al., 2013). Using exactly the same allylnitrile intoxication protocol and behavioral test battery, Saldana-Ruiz and colleagues observed significant group differences in vestibular rating scores at all times post-dosing (i.e., after 3, 6, 9, 12, 15, 18, and 21 days). At end-point functional testing, they observed five out of eight highly affected mice with high vestibular rating scores (>17), whereas the other three allylnitrile-treated mice remained largely unaffected with rating scores in the control range (<3). Similar as in our study, there was a high variability in observations, with affected and largely unaffected animals. However, in contrast to our study, affected mice did not recover to normal control-like behavior over time, but remained behaviourally impaired. Additionally, several other studies in both rats and mice have evaluated allylnitrile-induced effects on the peripheral vestibular system using VDR scores (Zang et al., 1999; Balbuena and Llorens, 2001; Tanii et al., 2004; Boadas-Vaello et al., 2005). However, in contrast to our study, allylnitrile dosing timing and selection were often distinctively different, and their protocols did not comprise TDCE co-treatment, which complicates direct comparisons. Nonetheless, all these studies obtained more or less consistent results, with animals exhibiting moderate to severe behavioral abnormalities following allylnitrile intoxication. The results of this study thus indicate major differences in the ototoxic effects of allylnitrile on the vestibular system of the CBACa and 129S1 mouse strains. In the CBACa transgenic mouse strain that we used in our study, preservation of vestibular function, and most likely also HCs in the vestibular neurosensory epithelia, seem to be more resistant to allylnitrile-induced ototoxic injury. One possible explanation for this decreased susceptibility of vestibular tissues, or increased selectivity for the cochlear organ, could be interstrain differences in vestibular susceptibility to allylnitrile. Certain mouse strains might be more sensitive to vestibular insults relative to others. However, at this point in time, ototoxic mechanisms are not yet sufficiently comprehended to identify reasons for interstrain differences in vestibular susceptibility. In their study, Saldana-Ruiz and colleagues chose to use the 129S1 strain since it was developed by Jackson as an adequate control of many genetically modified strains constructed on a 129/SV genetic background (Saldana-Ruiz et al., 2013). Since our experiment comprised ototoxic manipulations, in addition to the study of the auditory and vestibular sensory systems, the well-characterized CBACa was considered to be the most optimal background strain for this study (Ohlemiller et al., 2016).

### Effect of Allylnitrile on Hearing Function

#### Effect of Allylnitrile on Hearing Sensitivity

The results show that overall hearing sensitivity and cochlear OHC function of allylnitrile-treated mice were impaired across a wide frequency range relative to controls. Baseline ABR threshold values obtained for both groups were situated within the limits of normal hearing in mice (Zheng et al., 1999; Zhou et al., 2006) and, in agreement with previous findings, mice were found most sensitive to ABR stimulus frequencies of 16 kHz (Zheng et al., 1999; Reynolds et al., 2010). At 22- and 50 DPA, nearly all treated mice lacked detectable ABR thresholds at all frequencies tested (i.e, 2–32 kHz), with little preservation of hearing at 8 kHz in only 1 out of 6 subjects.

#### Effect of Allylnitrile on Outer Hair Cell Function

DPOAE thresholds were significantly elevated in the low-frequency region of the cochlea (i.e., 5–11 k Hz) and completely lacking in the mid-to high frequency region (i.e., 12–32 kHz). For both ABRs and DPOAEs, functional deficits remained permanent with no signs of recovery between both follow-up measurements. The co-occurrence of hearing loss and disrupted cochlear OHC function in the allylnitrile-treated mice indicates that the hearing loss observed in this study is likely to be due to damage to the sensorineural component of the auditory pathway, more specifically due to the loss of active amplifier function of the cochlear OHCs. This is not surprising, since the OHCs have been demonstrated to be the primary target of ototoxicity in the cochlea (Schacht et al., 2012). The loss of cochlear OHCs, as determined by DPOAEs, occurred in the typical basal-to-apical order characteristic for ototoxic injury, with complete functional loss at high frequencies and some preservation of cochlear OHC function at the lower frequencies. Nonetheless, nearly all mice were also found to be completely deaf at the low-frequency region of the cochlea (i.e., 2–8 kHz), as indicated by ABR thresholds.

#### Comparison to Other Species

To the best of our knowledge, the current study is the first in mice to examine the ototoxic effects of allylnitrile on hearing sensitivity and cochlear OHC function, providing new data on allylnitrile-induced cochleotoxicity. No previous data regarding allylnitrile-induced deficits on hearing and cochlear functions in mice are thus available for comparison purposes. In the rat, however, subacute dosing of allylnitrile has been demonstrated to produce similar results with undetectable ABR thresholds across all frequencies evaluated (Gagnaire et al., 2001). Additionally, many experimental studies in small mammals have shown a comparable loss of auditory functions after systemic exposure to other ototoxic agents (Murillo-Cuesta et al., 2010; Poirrier et al., 2010; Fernandez et al., 2019). The degree of hearing loss, however, is always heavily dependent on various factors, including the specific ototoxic drug, the treatment dose(s), the duration of use, and individual differences in genetic susceptibility (Murillo-Cuesta et al., 2010).

### Limitations

A limitation regarding the evaluation of vestibular function are to be noted in the present study. Our study, as well as the initial study performed by Saldana-Ruiz and colleagues (Saldana-Ruiz et al., 2012), did not include a comprehensive assessment of vestibular function. The various executed behavioral tests provided an indirect measure of peripheral vestibular function but did not directly evaluate the function of one or more otolith organs or semicircular canals. For more objective evaluation of vestibular function, recordings of quantitative measures, including the Vestibular Ocular Response (VOR), or vestibular evoked (myogenic) potentials, remain indispensable (Llorens et al., 2018).

Hearing extends up to 80–120 kHz in most strains of mice (Zheng et al., 1999; Ohlemiller et al., 2016). The limits of our hearing screening equipment were confined to the 32 kHz frequency level, leaving a fragile portion of the cochlea untested. However, ABR and DPOAE threshold testing for stimulus frequencies around the optimal regions of 8, 16, and 32 kHz are consistent with current state-of-the-art practices (Zheng et al., 1999; Kane et al., 2012). Second, due to equipment and software limitations, maximum sound stimulus intensities for ABR and DPOAE were limited to 80- and 70-dB SPL, respectively. If responses were completely absent at these frequency-specific stimulus intensity levels, artificial thresholds were considered to be 85 dB SPL for ABR and 80 dB SPL for DPOAE to enable subsequent analysis. It thus cannot be completely ruled out that allylnitrile-treated mice would display ABR and DPOAE thresholds at higher sound stimulus intensities.

### Conclusion

Our results demonstrate that isoflurane anesthesia significantly affects assessment of hearing function in mice, as determined by ABR and DPOAE, compared to ketamine/xylazine anesthesia. Despite the numerous advantages that make isoflurane an attractive option as a general anesthetic in murine auditory research, caution should be taken due to its confounding effects on the assessment of hearing function. Systemic co-treatment of CBACa transgenic mice with allylnitrile and TDCE resulted in a severe and permanent loss of hearing indicated by absent ABR thresholds at all frequencies tested. This hearing loss was accompanied by a significant loss of active amplifier function of the cochlear OHCs, most likely making it, as determined by DPOAEs, the underlying cause of the observed pathology. Unexpectedly, behavioral abnormalities reflective of vestibular impairment were observable for a short period of time only following allylnitrile administration, with complete behavioral recovery and signs reflective of impaired vestibular function completely lacking at the end of functional testing.

## Data Availability Statement

The original contributions presented in the study are included in the article/Supplementary Material, further inquiries can be directed to the corresponding author.

## Ethics Statement

The animal study was reviewed and approved by Ethics Committee for Animal Experiments of the University of Antwerp.

## Author Contributions

DV: conception and design of the study, conducting experiments, interpretation of data, and writing the manuscript. SE: conducting experiments, interpretation of data, data analysis, and writing the manuscript. DVD, PD, and OV: interpretation of data and revising the manuscript. KS: conducting experiments and collecting data. VS: providing insights into different effects of anesthesia and revising the manuscript. PP and VV: obtaining funding, conception and design of the study, supervision of the project, project management, and revising and drafting of the manuscript. All authors contributed to the article and approved the submitted version.

## Conflict of Interest

The authors declare that the research was conducted in the absence of any commercial or financial relationships that could be construed as a potential conflict of interest.
