# Supplementary material for: Effect of Oral Allylnitrile Administration on Cochlear Functioning in Mice Following Comparison of Different Anesthetics for Hearing Assessment
Source: Front Toxicol. 2021 Feb 25;3:641569. doi: 10.3389/ftox.2021.641569 (PMC8915850; doi:10.3389/ftox.2021.641569)
Supplement: Supplementary file 1 [file Data_Sheet_1.docx]

Supplementary Material

# Supplementary Figures and Tables

**Supplementary Table S1A.** Linear mixed models (LMMs) were fitted for each frequency separately to test for significant time x treatment interactions. Mann-Whitney U tests were performed for all frequencies at each point in time separately to test for differences in ABR thresholds between the allylnitrile (n=6) and control (n=5) group (baseline, 22DPA, and 50DPA). Significant values (p≤0.05*)* are shown in bold.

| **Calculated P-values for ABR Thresholds** | | | | | |
| --- | --- | --- | --- | --- | --- |
|  | Time*Treatment interaction | | Baseline  ALN vs. CON | 22DPA Threshold shift  ALN vs. CON | 50DPA Threshold shift  ALN vs. CON |
|  | F_2,18_ | P-value |  |  |  |
| 2kHz | 33,859 | **7,94 x 10^-7^** | 0,728 | **0,005** | **0,005** |
| 4kHz | 24,586 | **7,13 x 10^-6^** | 0,227 | **0,009** | **0,007** |
| 8kHz | 49,140 | **5,10 x 10^-8^** | 0,629 | **0,006** | **0,006** |
| 16kHz | 115,53 | **5,38 x 10^-11^** | 0,498 | **0,006** | **0,005** |
| 32kHz | 39,894 | **2,39 x 10^-7^** | 0,778 | **0,006** | **0,006** |

**Supplementary Table S1B.** LMMs were fitted for each frequency separately to test if there were significant differences in ABR threshold within each treatment group across all three points in time. Due to significant time effects in allylnitrile-treated mice, posthoc analysis with Tukey’s correction for multiple testing was carried out for pairwise comparisons of ABR thresholds between all three points in time. R software does not calculate p-values below 2 x 10^-16^. Significant values (p≤0.05*)* are shown in bold.

| **Calculated P-values for ABR Thresholds** | | | | | |
| --- | --- | --- | --- | --- | --- |
|  | Time effects  CON | Time effects  ALN | ALN  Baseline  vs. 22DPA | ALN  Baseline  vs. 50DPA | ALN  22DPA  vs. 50DPA |
| 2kHz | 0,081 | **6,31 x 10^-6^** | **< 2 x 10^-16^** | **< 2 x 10^-16^** | 1 |
| 4kHz | 0,081 | **6,23 x 10^-6^** | **< 2 x 10^-16^** | **< 2 x 10^-16^** | 1 |
| 8kHz | 0,240 | **2,82 x 10^-6^** | **< 2 x 10^-16^** | **< 2 x 10^-16^** | 1 |
| 16kHz | 0,361 | **2,13 x 10^-8^** | **< 2 x 10^-16^** | **< 2 x 10^-16^** | 1 |
| 32kHz | 0,321 | **2,64 x 10^-6^** | **< 2 x 10^-16^** | **< 2 x 10^-16^** | 1 |

**Supplementary Table S2A.** Linear mixed models (LMMs) were fitted for each frequency separately to test for significant time x treatment interactions. To test for differences in ABR thresholds between the allylnitrile (n=6) and control (n=5) group, Mann-Whitney U tests were performed for all frequencies at each point in time separately (baseline, 22DPA, and 50DPA). Significant values (p≤0.05*)* are shown in bold.

| **Calculated P-values for DPOAE Thresholds** | | | | | |
| --- | --- | --- | --- | --- | --- |
|  | Time*Treatment interaction | | Baseline  ALN vs. CON | 22 DPA  Threshold shift  ALN vs. CON | 50 DPA  Threshold shift  ALN vs. CON |
|  | F_2,18_ | P-value |  |  |  |
| 5278Hz | 23,800 | **8,82 x 10^-6^** | 0,086 | **0,005** | **0,03** |
| 6062Hz | 8,176 | **0,00298** | 0,395 | 0,060 | **0,011** |
| 6964Hz | 25,128 | **6,17 x 10^-6^** | 0,748 | **0,005** | **0,007** |
| 8000Hz | 13,740 | **0,000238** | 0,479 | **0,033** | **0,012** |
| 9189Hz | 40,896 | **2,02 x 10^-7^** | 0,479 | **0,004** | **0,006** |
| 10556Hz | 32,4298 | **1,08 x 10^-6^** | 1 | **0,004** | **0,005** |
| 12125Hz | 74,150 | **2,04 x 10^-9^** | 0,833 | **0,005** | **0,005** |
| 13929Hz | 274,024 | **3,32 x 10^-14^** | 0,465 | **0,002** | **0,003** |
| 16000Hz | 324,701 | **7,55 x 10^-15^** | 0,361 | **0,002** | **0,002** |
| 18379Hz | 232,948 | **1,36 x 10^-13^** | 0,0578 | **0,003** | **0,002** |
| 21112Hz | 66,158 | **5,06 x 10^-9^** | 0,833 | **0,005** | **0,005** |
| 24251Hz | 81,944 | **9,10 x 10^-10^** | 1 | **0,002** | **0,003** |
| 27858Hz | 38,233 | **3,31 x 10^-7^** | 0,724 | **0,004** | **0,005** |
| 32000Hz | 65,010 | **5,81 x 10^-9^** | 0,383 | **0,004** | **0,005** |

**Supplementary Table S2B.** LMMs were fitted for each frequency separately to test if there were significant differences in DPOAE threshold within each treatment group across all three points in time. Due to significant time effects in allylnitrile-treated mice, posthoc analysis with Tukey’s correction for multiple testing was carried out for pairwise comparisons of DPOAE thresholds between all three points in time. R software does not calculate p-values below 2 x 10^-16^. Significant values (p≤0.05*)* are shown in bold.

| **Calculated P-values for DPOAE Thresholds** | | | | | |
| --- | --- | --- | --- | --- | --- |
|  | Time effects  CON | Time effects  ALN | ALN  Base vs. 22DPA | ALN  Base vs. 50DPA | ALN  22DPA vs. 50DPA |
| 5278Hz | **0,005** | **2,58 x10^-5^** | **< 1 x 10^-5^** | **< 1 x 10^-5^** | 0.867 |
| 6062Hz | 0,081 | **0,0008** | **2 x 10^-5^** | **< 1 x 10^-5^** | 0.819 |
| 6964Hz | 0,410 | **3,22 x 10^-6^** | **< 1 x 10^-5^** | **< 1 x 10^-5^** | 0.882 |
| 8000Hz | 0,759 | **0,0005** | **< 1 x 10^-5^** | **< 1 x 10^-5^** | 0.961 |
| 9189Hz | 0,062 | **1,96 x 10^-8^** | **< 1 x 10^-5^** | **< 1 x 10^-5^** | 0.759 |
| 10556Hz | 0,240 | **8,59 x 10^-6^** | **< 2 x 10^-16^** | **< 2 x 10^-16^** | 1 |
| 12125Hz | 0,759 | **3,20 x 10^-9^** | **< 2 x 10^-16^** | **< 2 x 10^-16^** | 1 |
| 13929Hz | 0,410 | **3,15 x 10^-11^** | **< 2 x 10^-16^** | **< 2 x 10^-16^** | 1 |
| 16000Hz | 0,240 | **3,13 x 10^-158^** | **< 2 x 10^-16^** | **< 2 x 10^-16^** | 1 |
| 18379Hz | 0,410 | **7,20 x 10^-11^** | **< 2 x 10^-16^** | **< 2 x 10^-16^** | 1 |
| 21112Hz | 0,759 | **3,20 x 10^-9^** | **< 2 x 10^-16^** | **< 2 x 10^-16^** | 1 |
| 24251Hz | 0,178 | **4,60 x 10^-181^** | **< 2 x 10^-16^** | **< 2 x 10^-16^** | 1 |
| 27858Hz | 0,806 | **2,56 x 10^-8^** | **< 2 x 10^-16^** | **< 2 x 10^-16^** | 1 |
| 32000Hz | 0,036 | **8,42 x 10^-8^** | **< 2 x 10^-16^** | **< 2 x 10^-16^** | 1 |
